# Caregiver-led versus therapist-led training programme for caregivers of children with cerebral palsy in rural Malawi: a feasibility randomised controlled trial

**DOI:** 10.1186/s12887-026-07286-4

**Published:** 2026-07-07

**Authors:** Takondwa Connis Bakuwa, Gillian Saloojee, Wiedaad Slemming

**Affiliations:** 1 Department of Rehabilitation Sciences, Kamuzu University of Health Sciences, Blantyre, Malawi; 2 Division of Community Paediatrics, Department of Paediatrics and Child Health, Faculty of Health Sciences, University of Witwatersrand , Johannesburg, South Africa; 3 Department of Physiotherapy, Faculty of Health Sciences, University of Witwatersrand, Johannesburg, South Africa; 4 Children’s Institute, Department of Paediatrics and Child Health, Faculty of Health Sciences, University of Cape Town, Cape Town, South Africa

**Keywords:** Feasibility study, Cerebral palsy, Caregiver training, Caregiver-led programme, Rural

## Abstract

**Background:**

Caregiver-led interventions for children with cerebral palsy are increasingly implemented in resource-limited settings to improve access to services, yet evidence on their feasibility and effectiveness remains limited. This study assessed the feasibility of a group-based knowledge and skills caregiver-led training programme and compared its outcomes to a therapist-led training programme in rural Malawi.

**Methods:**

A two-arm feasibility randomised controlled trial was conducted in rural Mangochi, Malawi between January and August 2023, involving 83 primary caregivers of children with cerebral palsy aged 9 months to 14 years. Participants were randomised 1:1 to caregiver-led or therapist-led arms and received weekly practical knowledge and skills training sessions over seven weeks using the Malamulele Onward Carer-2-Carer Training Programme. Feasibility outcomes included recruitment, randomisation, retention, and fidelity of programme delivery. The primary outcome was caregiver quality of life measured using the Paediatric Quality of Life Family Impact Measure. Child outcomes included activity and participation measured using the Pediatric Evaluation of Disability Inventory and the Child and Adolescent Scale for Participation. Data were analysed using Stata 16.1 (Stata Corporation, College Station, Texas, USA). Outcomes are presented as medians and interquartile ranges due to some skewed distributions.

**Results:**

Thirty-nine of 42 and 37 of 41 caregiver–child pairs in the caregiver-led and therapist-led arms, respectively, completed baseline assessments and commenced the trial. Thirty-seven pairs per arm completed endline assessments and were included in the primary analysis, yielding a 97% completion rate. Programme delivery fidelity was 100% across both arms.

Improvements were observed in both caregiver- and child-level outcomes, with no evidence of differences between groups. For the primary outcome, the median change in Paediatric Quality of Life Family Impact Measure score was 9.7 in both arms (median difference 0; 95% CI: −5.0 to 5.0). For child outcomes, the median change in Pediatric Evaluation of Disability Inventory was 24 (11, 38) in the caregiver-led arm and 27 (6, 35) in the therapist-led arm, with a median difference of − 3 (95% CI: −19.0 to 13.0).

**Conclusion:**

The caregiver-led training programme is feasible and associated with improvements in caregiver and child outcomes, with no evidence of differences compared to therapist-led delivery. These findings support progression to a fully powered non-inferiority trial. If effectiveness and non-inferiority are confirmed, this approach may offer a promising task-sharing strategy for rehabilitation services in similar low-resource settings.

**Trial registration:**

The Pan African Clinical Trials Registry (PACTR202306542386770), retrospectively registered on 05 June 2023(https//pactr.samrc.ac.za/TrialDisplay.aspx? TrialID=25586).

**Supplementary Information:**

The online version contains supplementary material available at https://doi.org/10.1186/s12887-026-07286-4.

## Introduction

Cerebral Palsy (CP) is a complex lifelong childhood-onset neurodevelopmental disorder characterised by impaired movement and posture that cause limitations in activity and participation [1]. Evidence from population-based CP registers indicates that children in low- and middle-income countries (LMICs) experience both a higher prevalence of CP and greater severity of functional limitations compared with those in high-income settings [2, 3]. Reported prevalence estimates reach up to 3–5 per 1000 live births, and a high proportion of the children require substantial assistance for mobility and daily care [2, 3]. These patterns are commonly linked to preventable perinatal risk factors, delayed diagnosis, limited access to early intervention, and restricted availability of ongoing rehabilitation services [2, 4, 5]. As a result, children with CP in LMICs are more likely to experience significant impairments in movement, activity, and participation across the lifespan [4, 6, 7].

The complexity of CP can present significant challenges for caregivers, particularly in rural settings where access to rehabilitation services is limited. In this study, caregivers refer specifically to family members, most often mothers or grandmothers, who assume primary responsibility for the daily care of children with CP despite having limited formal training or resources [8, 9]. Because these caregivers are responsible for implementing day-to-day care and therapeutic activities in the home, the effectiveness of rehabilitation is closely dependent on their knowledge, skills, and confidence. This reality underscores the need for accessible caregiver skills training programmes that are responsive to the contextual and practical challenges faced by families in resource-constrained environments.

Caregiver skills training programmes refer to structured interventions designed to equip caregivers with practical knowledge and skills to support their children’s daily functioning [9, 10]. Evidence from largely qualitative studies indicates that such programmes are associated with improvements in caregiver well-being, including reduced stress, improved confidence, and enhanced coping, alongside gains in caregiving competence [10–12]. Central to these benefits is caregiver self-efficacy, defined as a caregiver’s belief in their ability to successfully manage caregiving tasks and challenges [13]. Higher self-efficacy has been linked to greater engagement in home-based care activities, more consistent use of therapeutic strategies, and improved caregiver well-being, making it a relevant outcome for caregiver skills training interventions [13, 14].

Caregiver skills training typically includes practical strategies to support children’s active participation in everyday activities, such as safe positioning, feeding and communication strategies, play-based stimulation and problem-solving during daily routines [11, 12, 15, 16]. Many programmes also incorporate components addressing caregiver well-being and mental health, including stress management, acceptance-based coping strategies, and peer support [12], some draw explicitly on principles from ‘Acceptance and Commitment Therapy’ to support psychological flexibility and resilience [17, 18]. By strengthening caregivers’ capacity to embed therapeutic strategies into everyday activities, these programmes enable more consistent practice in the home environment, which in turn supports children’s functional performance and participation in family life [15, 19]. Moreover, these practical ideas reduce caregiver stress and promote good mental health [12] In LMIC settings such as Malawi, where access to specialist rehabilitation services is limited, caregiver skills training represents a feasible and evidence-informed approach to extend rehabilitation beyond clinic-based care.

In LMICs, task-sharing strategies have emerged as a pragmatic response to workforce shortages in rehabilitation, aiming to reduce inequalities in access to care [20]. These approaches enable non-specialists to deliver interventions to families of children with neurodevelopmental disabilities [20, 21]. Several studies involving caregivers of children with neurodevelopmental disorders have explored the feasibility and effectiveness of the delivery of skills training by non-specialists including teachers and community volunteers [21–23]. Caregivers involved in these skills training programmes have reported improved knowledge, skills and reduced stress [10, 21, 22], as well as improvements in the neurodevelopmental outcomes of their children [22].

In this study, the caregiver-led intervention is delivered by trained expert caregivers who act as the primary facilitators of a structured rehabilitation training programme. Expert caregivers, parents or family members with lived experience of caring for a child with CP, deliver standardised training sessions focused on functional skill acquisition, problem-solving in daily routines, and guided practice of therapeutic activities within the home environment. Physiotherapists provide initial training, supervision, and quality assurance but do not directly deliver sessions to participants, enabling a clear comparison with therapist-led training delivery.

This approach explicitly recognises the potential value of engaging expert caregivers: trained parents or family members with lived experience, in delivering skills training for caregivers of children with neurodevelopmental conditions [23–25]. In this approach, expert caregivers are positioned as programme facilitators who deliver structured training content aimed at strengthening caregivers’ practical caregiving skills, confidence, and problem-solving abilities, with downstream effects on child functioning and caregiver-child interaction [9, 10]. This represents a shift from their earlier roles in peer counselling and psychosocial support [19] toward more structured roles in programme delivery. While recent programmes such as the Baby Ubuntu and Akwenda programmes in Uganda and the ongoing LEAP-CP trial in several LMICs have incorporated caregiver-led components, these were embedded within multi-component programmes [23, 24, 26]. As such, the caregiver-led delivery approach has not yet been evaluated independently, making it difficult to determine its standalone feasibility and contribution to observed outcomes. This gap is especially relevant in resource-limited contexts, where engaging expert caregivers may serve as a practical and sustainable form of task sharing within rehabilitation services.

There is therefore a compelling need to conduct a powered trial to assess whether a caregiver-led training programme can serve as a viable alternative to therapist-led training programmes in LMIC settings. Such an evaluation would ultimately require a non-inferiority design to determine whether a community-embedded, caregiver-led approach can achieve outcomes comparable to standard therapist-led models within an acceptable margin [27]. However, the design of a powered non-inferiority trial depends on robust preliminary evidence on feasibility parameters, including recruitment and retention rates, fidelity of implementation, sustained caregiver engagement, and the feasibility of core trial procedures such as randomisation, follow-up, and outcome data collection in rural, low-resource settings [28, 29]. Establishing these parameters is essential for the design of a future non-inferiority trial to evaluate caregiver-led delivery as a potential alternative to therapist-led approaches.

Therefore, to address this gap, we conducted a feasibility randomised controlled trial in a rural Malawian setting to explore the delivery of a caregiver-led training programme and to compare its outcomes with a therapist-led training programme. The objectives were to assess:

(i) the feasibility of recruiting, randomising, and retaining caregiver-child pairs;
(ii) the completion rate of the programme in both arms;
(iii) the fidelity of programme delivery by facilitators on both arms and.
(iv) preliminary patterns of caregiver- and child-level programme outcomes.

## Materials and methods

This study is reported in accordance with the Consolidated Standards of Reporting Trials (CONSORT) extension for pilot and feasibility randomised controlled trials.

### Design

A seven-week, two-arm, assessor-blinded feasibility randomised controlled trial was conducted in a rural area of Mangochi district in Malawi between January and August 2023. The trial included a four-month preparatory period; two weeks of baseline assessments; seven weeks of programme delivery (one session per week); followed by a four-week home practice phase during which caregivers implemented the skills learned within their usual daily routines; and two weeks of post-programme assessments. During the home practice period, caregivers did not receive supervised home visits; however, informal follow-up and support occurred remotely through existing community structures, with sub-centre leaders checking in with caregivers during routine weekly meetings outside the training programme. This approach was intended to reflect real-world conditions in resource-constrained settings and to assess the feasibility of skill application without intensive professional supervision.

### Setting

The study took place in Namwera, a rural community in the Mangochi District, one of the largest districts in southern Malawi. The study site was Tiyende Pamodzi, a Community-Based Organisation (CBO) providing essential services to children with disabilities in Namwera. It partners with the Mangochi Government District Hospital to offer medical and rehabilitation care to the children on its registry. In 2023, the centre had a register of over 400 children with physical impairments, most of whom were suspected to have cerebral palsy (CP). Similar to other rural district hospitals in Malawi, Mangochi District Hospital lacks sufficient physiotherapists and rehabilitation assistants, which limits its ability to provide services to remote areas like Namwera.

### Study participants

Trial participants were primary caregivers of children aged 9 months to 14 years with a confirmed diagnosis of cerebral palsy, who were receiving rehabilitation services from the Tiyende Pamodzi group and who provided informed consent to participate in the study. In this study, the primary caregiver refers to any family caregiver involved in the primary care of the child with CP who can be their mother, father, aunt, uncle, grandparent or sibling [30, 31].

Caregivers who were unwilling or unable to commit to completing the full seven-week training were excluded. Moρeoveρ, caregivers with severe cognitive, hearing, or communication impairments that would preclude meaningful participation in group training and assessments or make participation unsafe were excluded. In addition to this, caregivers currently participating in another structured CP-related training or intervention programme were also excluded to avoid contamination of outcomes. Details of recruitment are further described in a subsequent section.

### Screening, recruitment, and randomisation

Screening was conducted over 10 days using the Tiyende Pamodzi CBO records of children with disabilities across eight subcentres. Records were reviewed to identify children presenting with clinical features suggestive of CP, based on documented developmental history, motor delay, abnormal posture or movement patterns, and known perinatal or early-life risk factors for CP [32]. Where uncertainty existed, children were considered to have a “clinically suspected diagnosis” pending specialist review. A paediatric neurologist from Zomba Central Hospital (approximately 160 km from the study site) reviewed the clinical records of the identified children and conducted physical clinical assessments to confirm the diagnosis of CP prior to their enrollment into the study.

Following confirmation of eligibility, primary caregivers of children with confirmed diagnosis of CP were approached by a trained research assistant. The assistant provided verbal and written information about the study aims, procedures, potential risks, and benefits, allowed adequate time for questions, and obtained written informed consent prior to participation. After completion of baseline assessments, participants were individually randomised to the intervention delivery arms using a computer-generated randomisation sequence. Allocation was concealed from outcome assessors to minimise bias.

The participants were then randomly allocated in a 1:1 ratio to the caregiver-led and therapist-led arms of the programme. Randomisation was conducted by a statistician independent of the trial team using Stata 16.1 (Stata Corporation, College Station, Texas, USA). Participants were stratified according to the child’s level of functional ability, classified using the Gross Motor Function Classification System (GMFCS levels I–V) [33], to ensure balanced representation of functional severity across trial arms. Within each GMFCS stratum, participants were randomised in a 1:1 ratio to the intervention delivery groups using a computer-generated random sequence. Allocation occurred at the individual level following completion of baseline assessments.

### Sample size

As this was a feasibility study, the primary aim was not to test for statistically significant differences between the two intervention groups, but to assess key feasibility parameters such as recruitment, retention, and to explore the pattern and variability of programme outcomes. A target sample size of 70 participants was selected based on recommendations for external pilot trials, as this number is considered sufficient for reliably estimating standard deviations to inform the design of a future powered trial [34]. To allow for potential dropouts, the target was increased to 80 participants. Ultimately, 83 participants were recruited, slightly exceeding the planned sample, which further strengthens the reliability of feasibility estimates.

**Fig. 1:**
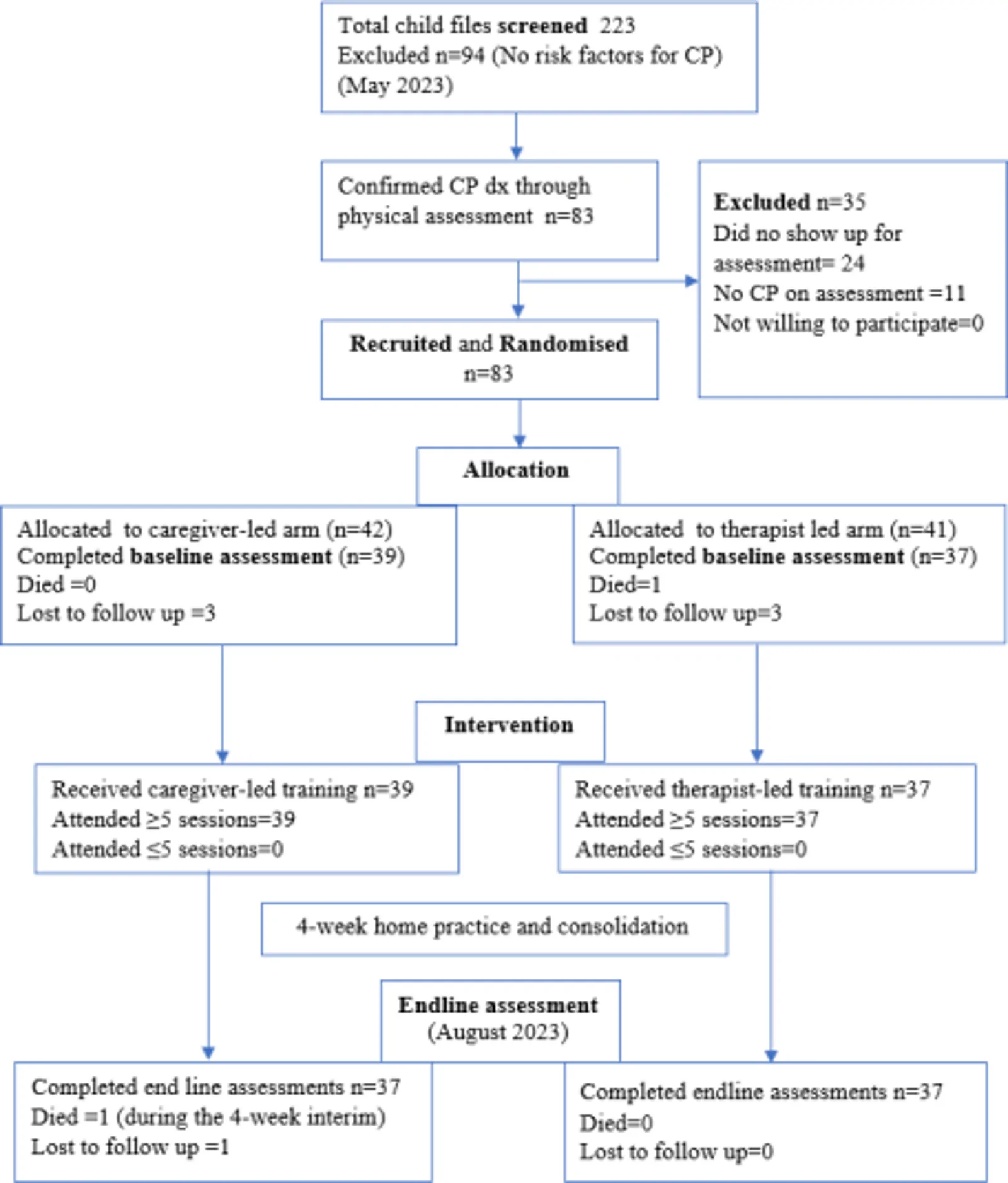
The flow of participants in the study. Legend: CONSORT flow diagram summarising participant screening, eligibility assessment, randomisation, allocation to caregiver-led or therapist-led training arms, follow-up, and inclusion in the final analysis

Out of the 83 caregiver-child pairs recruited into the study, 76 completed baseline assessment and entered the trial and 74 completed the programme and endline assessments, with 37 pairs in each arm. Figure 1 shows the flow of the participants through the study.

### Programme preparation and facilitator training

**Fig. 2:**
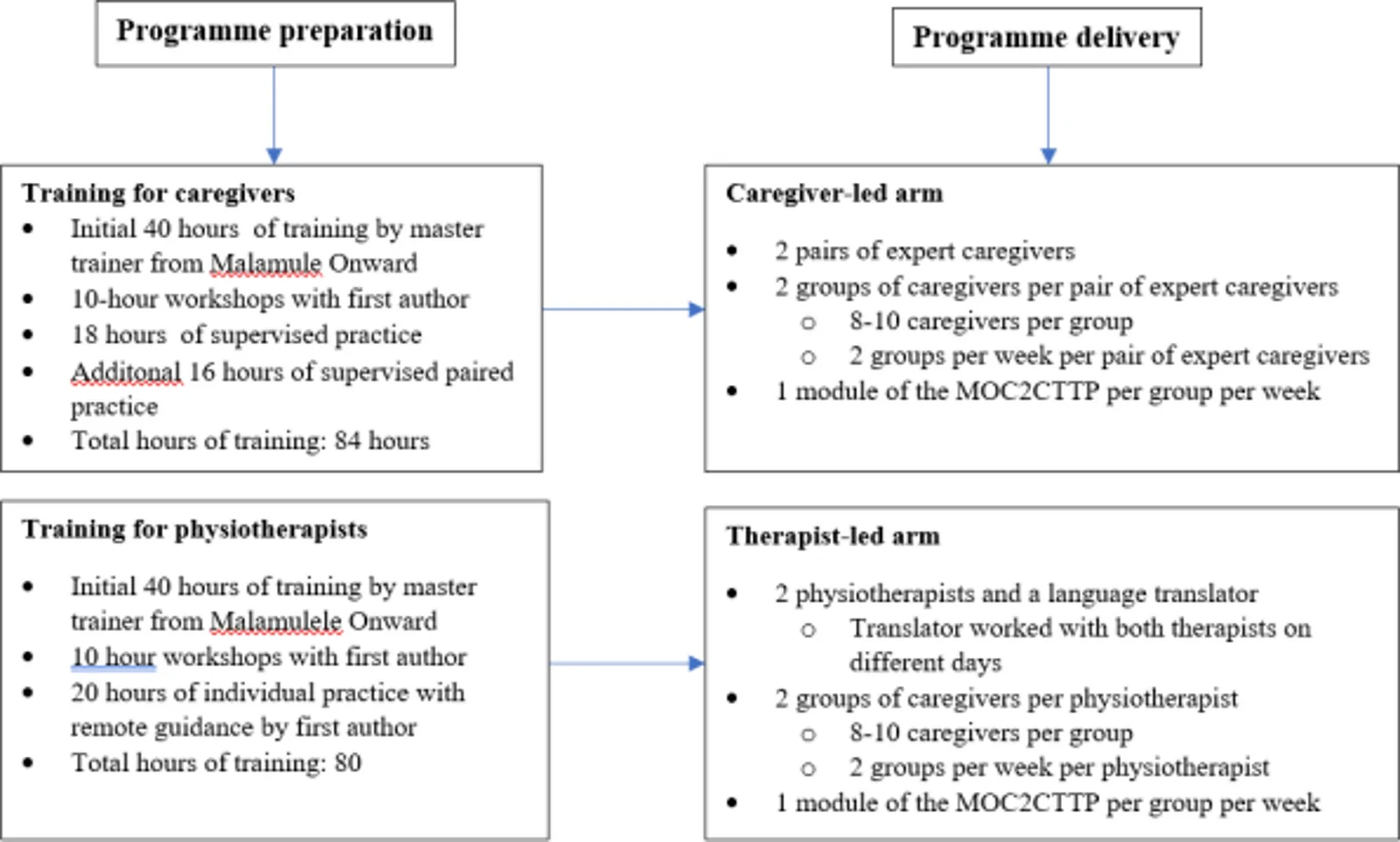
Programme preparation and delivery across trial arms. Legend: This schematic illustrates the preparatory training and delivery structure of the Malamulele Onward Carer-to-Carer Training Programme (MOC2CTP). The left panels summarise facilitator preparation, including total training hours and supervision, for expert caregivers and physiotherapists. The right panels depict programme delivery for the caregiver-led and therapist-led arms, including group size, facilitator configuration, session frequency, and module dosage. Programme content, materials, session format, and dosage were standardised across arms; the primary distinction was the background of the programme facilitator

Programme preparation preceded intervention delivery and is summarised in Fig. 2. Ten caregivers were purposively identified by CBO leadership to attend initial training with a master trainer based on caregiving experience, engagement with the CBO, and leadership qualities. Following this training, four were selected as expert caregivers based on facilitation and communication skills, alongside two physiotherapists working with children with CP at the CBO and District Health Office. All six facilitators completed structured training anchored in the Malamulele Onward Carer-to-Carer Training Programme (MOC2CTP), beginning with an intensive five-day workshop led by the master trainer from Malamulele Onward, during which the full curriculum was demonstrated [35].

This initial training was followed by additional workshops, supervised practice, and team facilitation coordinated by the first author, tailored to the roles of expert caregivers and physiotherapists. The scope of practice for the expert caregivers was also clarified during these workshops. Training activities focused on familiarisation with programme content, experiential delivery methods, co-facilitation skills, and preparation of low-cost materials for use during the programme. Modifications proposed during the initial training with the master trainer, including extending the delivery time for modules and restructuring the ‘eating and drinking’ module, were implemented. Details of these modifications are reported elsewhere [35, 36]. An additional period of team facilitation training was introduced to strengthen paired delivery among expert caregivers based on observations during practice time .The total duration and structure of facilitator preparation are detailed in Fig. 2.

An executive member from the CBO was trained as a Yao language interpreter for the two physiotherapists, who only spoke Chichewa language. He attended preparation trainings to familiarise himself with the programme content and terminology. Two health surveillance assistants from the Namwera Community Health Centre were trained to monitor programme delivery by the physiotherapists and expert caregivers in terms of duration of sessions and completion of module elements. They were oriented to the programme during facilitator practice workshops and trained to use two monitoring checklists (Additional files 1 and 2).

### Intervention

Participants in each arm participated in a caregiver skills training programme, the MOC2CTP. This programme, developed by Malamulele Onward non-profit organisation in South Africa, comprises seven modules, each targeting specific practical knowledge and skill areas related to CP (Table 1). The modules are designed to be delivered by a pair of trainers who themselves are caregivers of children with CP. Each session includes an ice-breaker activity based on themes of psychosocial support; a second section on the main topic of discussion, followed by a larger section of practical experiential demonstrations. The programme uses a picture-based manualised guide and accessible everyday materials such as plastic sheets, feeding utensils, blankets, pillows, balls and food. Each module was designed to be delivered in one session lasting 2 to 2.5 h. However, during training preparation, extension of module delivery time was recommended to accommodate caregivers with diverse educational backgrounds. The average session lengths during the trial are presented under the results section.

**Table 1:** Content of the Malamulele Onward Carer-2-Carer Training Programme

Module	Content
1	Introduction to Cerebral Palsy: What is it and how does it affect my child
2	Cerebral Palsy as a way of life: Looking after my child throughout the day
3	Getting active: Getting my child’s body ready to move throughout the day
4	Eating and drinking: making meal times safe and comfortable for my child
5	Communication: My child and I understand each other
6	Cerebral Visual Impairment: Understanding where and what my child can see
7	Play: Unlocking my child’s potential

This table outlines the seven core modules of the Malamulele Onward Carer-2-Carer Training Programme. The content is designed to equip caregivers of children with cerebral palsy with practical knowledge and skills to support their child’s daily functioning, communication, and overall well-being in a home setting. Further information about the programme can be found through the Malamulele Onward website: https:www.cpchildren.org/what-we-offer/carer-2-carer-programme

Caregivers attended one group session per week over seven consecutive weeks. Participants decided on optimal weekly meeting times, and session activities included a communal lunch break, where they shared meals and fed their children. Sessions followed an identical structure across both arms and covered one module per session, allowing time for practice and consolidation between sessions. Delivery arrangements, group sizes and session frequency for each arm are illustrated in Fig. 2.

The primary distinction between trial arms was the background of the programme facilitator: modules were delivered either by trained expert caregivers working in pairs (caregiver-led arm) or by physiotherapists delivering modules individually with support from a trained interpreter (therapist-led arm). Programme content, materials and format were otherwise standardised across arms. The delivery structure in each arm reflected the intended real-world mode of implementation for that facilitator group (i.e. expert caregivers co-facilitating, while physiotherapists facilitating individually).

### Access to usual care

During the study period, all study participants continued to access the usual services from Tiyende Pamodzi CBO. The usual services included receiving individual goal-directed therapies delivered by a physiotherapist, assistive devices, nutritional supplements, and medication for seizure control and referrals to district and central hospitals for further multidisciplinary care. Details of other services obtained were documented during the trial.

### Data collection and outcome assessment

- Feasibility outcomes

Feasibility was assessed through recruitment, retention, and completion rates, alongside programme delivery fidelity, including adherence to session outlines and duration. Guidelines from the National Centre for Complementary and Integrative Health were used as a benchmark for feasibility which recommend setting retention and attendance targets to assess programme viability [37]. We aimed for at least 80% retention and 70% of participants attending 70% of sessions and [38]. The same represented our criteria for progressing to a powered trial.

Feasibility data were collected throughout programme delivery by two trained Health Surveillance Assistants (HSAs), independent of programme facilitation. Attendance and retention were monitored using session registers at each workshop. Programme delivery fidelity was assessed at every session using two structured monitoring checklists derived from the Malamulele Onward training package. These captured multiple fidelity domains, including adherence, dosage, quality, and differentiation [39].

The first checklist (Additional file 1) documented adherence and dosage by recording delivery of programme components and session duration, while the second checklist (Additional file 2) assessed delivery quality and differentiation across arms [39, 40]. All checklists were signed by the facilitator, a CBO executive member, and the implementation monitor to confirm completeness. HSAs were also trained to observe and report any unintended effects during delivery.

In this report, only the structured quantitative checklist items were summarised descriptively to assess feasibility and fidelity of delivery. Narrative responses captured within the checklists were analysed alongside in-depth interview data in a separate qualitative study and are reported elsewhere [36].

b) Programme outcomes

Programme outcome assessments were questionnaire-based and conducted pre- and post-intervention by two trained rehabilitation technicians who were blinded to trial allocation. Assessments were scheduled based on participants’ area of residence, irrespective of allocation, to preserve blinding. Each assessment session lasted approximately one hour and was conducted over two days per participant for quality control. Data collection for the full sample was completed over a two-week period, with quantitative data captured and managed using REDCap, hosted by the University of the Witwatersrand.

### Main programme outcome

The primary exploratory programme outcome was caregiver quality of life, measured using the Paediatric Quality of Life Family Impact Module (PedsQL-FIM). The PedsQL-FIM is a 36-item questionnaire assessing the impact of a child’s health condition on caregivers and families across six domains: physical, emotional, social, cognitive functioning, communication, and worry. Items are rated on a 5-point Likert scale (0–4), with higher scores indicating better quality of life. The instrument has demonstrated strong internal consistency (Cronbach’s α = 0.75–0.97) [41].

Outcomes were assessed at baseline and four weeks post-intervention to allow for home practice and consolidation, consistent with the PedsQL-FIM recall period of the “past month” [42]. Although the primary intent of the programme was to improve caregiver knowledge and skills, caregiver quality of life was selected as the main outcome because it captures the broader psychosocial and physical impact of caregiving, including stress, fatigue, and emotional burden. The PedsQL-FIM is a validated measure with strong psychometric properties and has been used in similar LMIC caregiver intervention studies, supporting meaningful cross-study comparison [43, 44].

### Other programme outcomes

Other caregiver-level programme outcomes included caregiver knowledge and self-efficacy, assessed using programme-specific questionnaires developed by Malamulele Onward. The caregiver knowledge questionnaire consists of 26 items covering core caregiving domains such as feeding, positioning, communication, and play. Each item is rated as “True,” “Not True,” or “Don’t Know,” with higher scores indicating greater caregiving knowledge. The self-efficacy questionnaire includes 13 items in which caregivers rate their confidence in performing specific caregiving tasks using a 10-point scale, from 1 (“not confident at all”) to 10 (“extremely confident”).

Although these tools have not undergone formal standardisation or psychometric testing, they have been refined over a period of seven years. Their development involved literature reviews and collaborative input from a multidisciplinary team, including a physiotherapist, occupational therapist, speech therapist, and an expert caregiver. Improvements were also informed by feedback gathered through caregiver interviews to ensure cultural and contextual relevance.

Child-level outcomes were assessed through caregiver-reported tools focusing on activity and participation. These included the Ugandan version of the Paediatric Evaluation of Disability Inventory (PEDI-UG) and the Child and Adolescent Scale of Participation (CASP), both of which have demonstrated strong reliability with correlation coefficients above 0.86 [45]. The PEDI assesses children aged 0.5–7 years and can be applied to older children with lower functional performance [46], while the CASP is validated for use across childhood and adolescence (3–18 years) [47].

These measures were selected for their relevance to functional performance and participation in children with disabilities in low-resource settings. PEDI-UG contains 185 items on a binary scale format (yes/no) which assess functional skills and caregiver assistance across self-care, mobility, social functioning, and communication, with higher scores indicating greater independence [45]. The CASP complements the PEDI-UG by capturing broader participation across home, school, community, and daily activities, rated on a four-point scale from “unable” to “full participation” [47, 48]. Because several domains were inapplicable to all children in this study, total scores could not be calculated. Therefore, only domain scores are reported.

All tools were translated into Chichewa and Yao. After obtaining permission and being granted access to the copyright by the owners of the tools, a team of clinical experts and social science linguists managed the translation through forward and backward translation, followed by cognitive testing and reconciliation with the 10 caregivers involved in the formative study [35].

### Data analysis

Statistical analysis was conducted using Stata 16.1 (Stata Corporation, College Station, Texas, USA). As this was a feasibility trial, the focus of analysis for the programme outcomes was on estimating the variability and patterns of outcomes rather than testing for statistical significance. Therefore, categorical and proportion-based outcome variables were summarised using frequencies and percentages. Continuous outcome variables were summarised using means and standard deviations (SD) for approximately normally distributed data, or medians and interquartile ranges (IQR) for skewed data. Changes between groups were described using mean or median differences with 95% confidence intervals.

The percentile bootstrap method was used to estimate the confidence intervals. This approach was selected for its robustness in small sample sizes and non-normal data, and because it allows reliable estimation of differences without relying on traditional parametric assumptions [49].

Missing outcome data were minimal and were not imputed. All analyses were conducted using a complete-case approach, whereby only participants with complete baseline and follow-up data were included in the bootstrap analyses. Formal imputation methods were not applied, given the feasibility focus of the study and the exploratory nature of the analyses.

## Results

### Description of the study sample

The primary caregiver of the majority (72%) of the children was their mother and most (70%) of the children aged ≥ 36 months were not attending creche/school. All the caregivers were primarily subsistence farmers, whilst a few (12%), also had a small business or wage employment. Further characteristics of the caregiver-child pairs are shown in Table 2.

**Table 2:** Characteristics of the study sample

Child characteristics	Caregiver-led arm *n* = 42	Therapist-led arm *n* = 41	Overall *N* = 83
**Age (months)**			
Median (IQR)	(9, 168)	54 [35,108]	50 [29, 90]
Range	46.5 [27,84]	(10, 156)	(9, 168)
**Sex**			
Female	15 (50%)	15 (50%)	30 (36%)
Male	27 (51%)	26 (49%)	53 (64%)
**GMFCS**			
Level 1 to III (ambulatory)	15(50%)	15(50%)	30(36%)
Level IV to V (non-ambulatory)	27(51%)	26(49%)	53(64%)
**Cerebral Palsy sub-type**			
Spastic	29 (48%)	32 (52%)	61 (73%)
Dyskinetic	13(59%)	9 (41%)	22 (27%)
**School-going status**			
≤ 36 months (underage for schooling)	17 (59%)	12 (41%)	29 (35%)
≥ 36 months but not in creche/school	20 (53%)	18 (47%)	38 (46%)
≥ 36 months and in creche/school	5 (31%)	11(69%)	16 (19%)
**Caregiver characteristics**			
Age (years) Median (IQR)	25.5 [22,33]	29 [23,40]	27 [22,38]
**Sex**			
Female	40 (51%)	38 (49%)	78 (94%)
Male	2 (40%)	3 (60%)	5 (6%)
**Relationship to child**			
Mother	34 (57%)	26 (43%)	60 (72%)
Father	2 (50%)	2 (50%)	2 (5%)
Grandmother	4 (27%)	11 (73%)	15 (18%)
Grandfather	0	1 (100%)	1 (1.2%)
Aunt/sister	2 (67%)	1 (33%)	3 (4%)
**Education level**			
None to Junior Primary (0-grade 4)	23(44%)	29(56%)	52 (63%)
Senior Primary (grade 5–8)	18(72%)	7(28%)	25 (30%)
Junior Secondary (grade 9–10)	1(17%)	5(83%)	6 (10%)
**Marital status**			
Single parent	11(44%)	14(54%)	25(30%)
Married	31(53%)	27(47%)	58(70%)
CBO Membership (months) Median (IQR)	24 [12, 63]	26 [12, 65]	26 [12, 63]
**Occupation/employment**			
Subsistence farming alone	37 (51%)	36 (49%)	73 (88%
Subsistence farming and wage labour	3 (43%)	4 (57%)	7 (8%)
Subsistence farming and small business	2 (67%)	1(33%)	3 (4%)

This table presents baseline characteristics of children with cerebral palsy and their caregivers across the caregiver-led and therapist-led arms of the study. Data are shown as counts and percentages for categorical variables, and medians with interquartile ranges (IQRs) and ranges for continuous variables

### Feasibility outcomes

#### Participant retention and programme completion

Seventy-six caregiver-child pairs completed baseline assessments by 30 May 2023, whilst 74 caregiver-child pairs completed post-programme assessments by 30 August 2023 (Fig. 1). This represents a 97% programme completion rate.

#### Fidelity of programme delivery

The implementation monitors captured a 100% adherence to the delivery of programme sections over all seven workshops of programme delivery by both groups of facilitators. All modules delivered in each arm covered all 13 to 17 components as outlined in the programme manual (Additional file 2). Sessions lasted an average of three hours and five minutes on the caregiver-led arm and an average of three hours and thirty-five minutes on the therapist-led arm, excluding 45-minute lunch breaks. Both physiotherapists required a language interpreter for each session which slightly increased session delivery time.

### Programme outcomes

#### Main programme outcome

Both arms showed improvements in the main outcome, with mean change scores of 8.3 (95% CI: 5.9, 10.7) in the caregiver-led group and 9.9 (95% CI: 7.0, 12.7) in the therapist-led group. The mean difference between the two groups was 1.5 (95% CI: -2.1 to 5.2). As the confidence interval includes zero, there is no evidence of a statistically significant difference between groups, suggesting comparable gains. Table 3 presents the detailed total scores and median scores for each sub-section of the PedsQL-FIM.

**Table 3:** Pre- and post-programme change in caregiver quality of life (PedsQL-FIM)

Outcome	Before training		After training		Post-intervention Between-group Median difference (95%CI)
	Caregiver-led arm Median (IQR *n* = 39	Therapist-led arm Median (IQR) *n* = 37	Caregiver-led arm Median (IQR) *n* = 37	Therapist-led arm Median (IQR) *n* = 37	
**Physical functioning**	58.3[50,75]	62.5[54.2,79.2 ]	62.5[58.3, 75]	75[66.3,83.3]	12.5(2.8, 22.2)
**Emotional functioning**	50[40,55]	55[40,60]	55[50,70]	65[60,70]	10(2.2, 17.8)
**Social functioning**	75[56.3, 100]	68.7[50, 87.5]	81.3[75,100]	81.3[68.8, 100]	0(-12.6, 12.6)
**Cognitive functioning**	80[50,90]	75[60,85]	85[70,100]	85[75,90]	0(-10.9, 10.9)
**Communication**	83.3[66.6,91.]	75[75,83.3]	83.3[75,100]	91.6[75,100]	8.3(-2.1, 18.7))
**Worry**	60[50,75]	70[55,75]	65[55,80]	75[60,80]	5(-5.6, 15.6)
**Daily Activities**	66.6[50,100]	58.3[41.6,83.3]	83.3[66.6,100]	83.3[66.6,91.6]	0(-17.3, 17.3)
**Family relationships**	90[65,100]	90[65,100]	100[75,100]	90[75,100]	-10(-24.3, 4.3)
**Total PedsQLFIM score**	66.9(± 12.5)	67.2 (± 14.3)	74.5(± 10.8)	77 (± 9.7)	1.5 (-2.1, 5.2)

This table presents changes in caregiver quality of life measured using the Paediatric Quality of Life Family Impact Measure (PedsQL-FIM) across multiple domains before and after the training programme, comparing the caregiver-led and therapist-led arms. Data are shown as medians with interquartile ranges (IQR). Between-group median differences are reported alongside their 95% confidence intervals. The total PedsQL-FIM score (*) is presented as a mean with a standard deviation. The mean difference is reported with a 95% CI. All estimates are descriptive change scores for feasibility purposes; no hypothesis testing was performed

#### Other programme outcomes

**Fig. 3:**
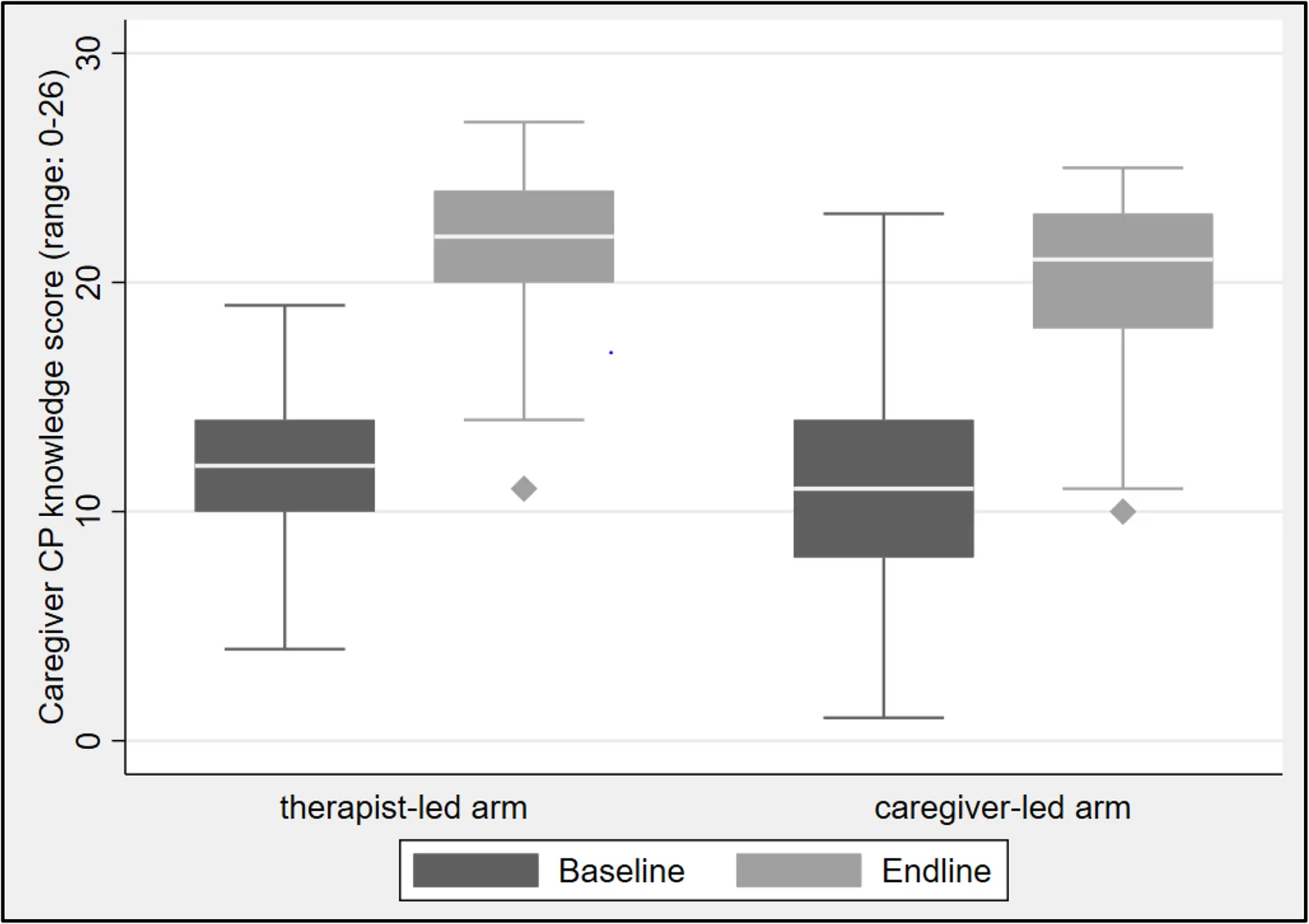
Pre- and post-implementation change in caregiver knowledge. Legend: Distribution of caregiver knowledge scores across the two groups before and after the training programme. The box plots display the median (horizontal line), interquartile range (box: 25th to 75th percentile), and minimum and maximum values (whiskers). Outliers are shown as individual dots.

The mean and standard deviation for the total knowledge scores at baseline were 11.3 ± 3.9 and 12.2 ± 2.9 on the caregiver-led and therapist-led arms respectively. This improved to 20 ± 3.7 and 21.4 ± 3.5 on the caregiver-led and therapist-led arm respectively. The mean difference between the two groups was − 2 (95% CI: -4.8, 0.8), indicating comparable gains. The distribution of knowledge scores before and after the training programme across the groups is illustrated in (Fig. 3).

**Fig. 4:**
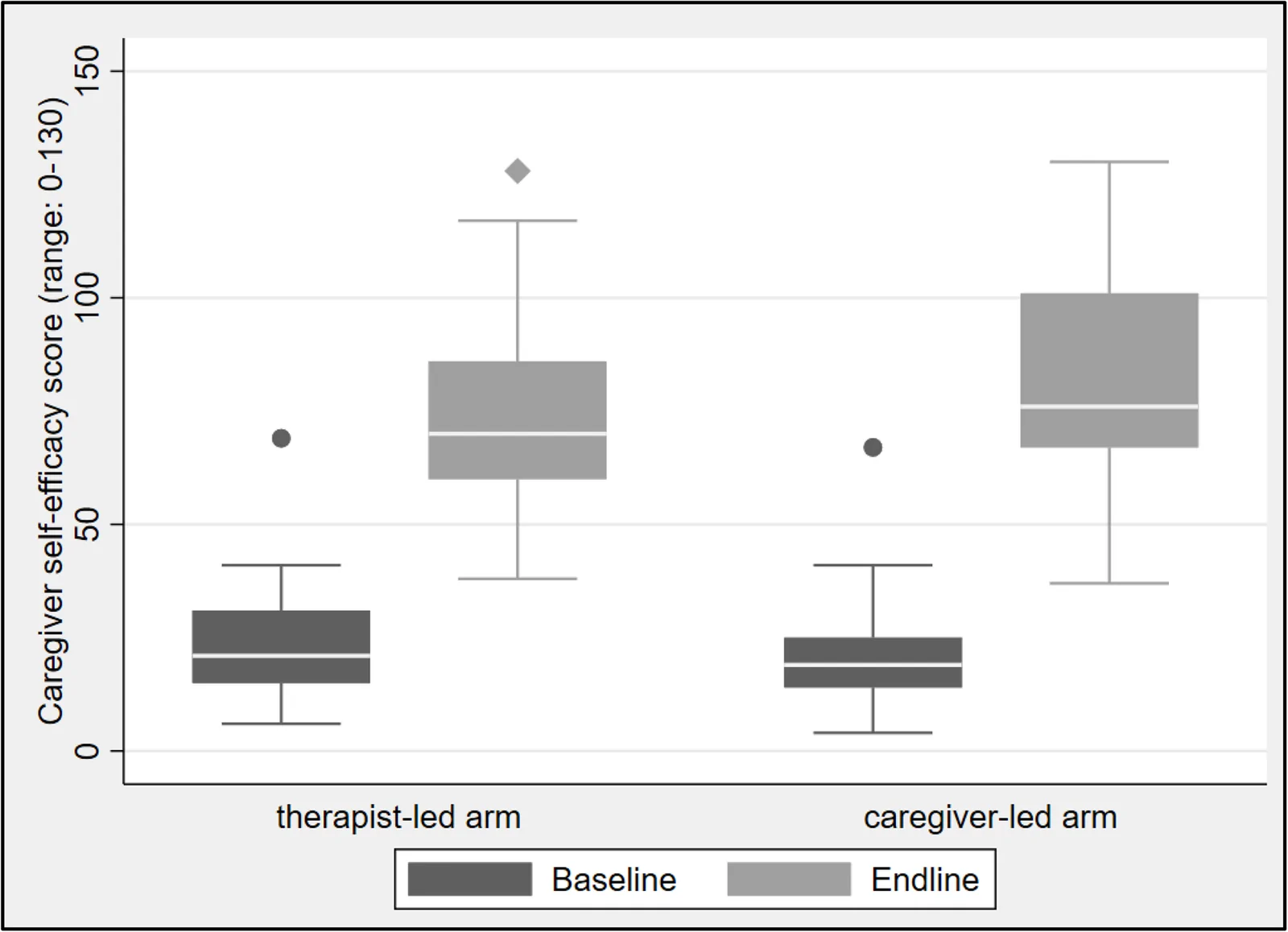
Pre- and Post- implementation change in caregiver self-efficacy. Legend: Distribution of caregiver self-efficacy scores across the two groups before and after the training programme. The box plots display the median (horizontal line), interquartile range (box: 25th to 75th percentile), and minimum and maximum values (whiskers). Outliers are shown as individual dots

The median self-efficacy scores and interquartile ranges at baseline were 19 (14, 25) and 21 (15, 31) on the caregiver-led and therapist-led arms respectively. The scores increased to 76 (67,101) and 70 (60, 86) on the caregiver-led and therapist-led arms respectively. The caregiver-led arm had a higher median self-efficacy score than the therapist-led arm, with a median difference of 9 points (95% CI: 1.1 to 19.1). (Fig. 4).

The median and interquartile ranges of the total Paediatric Evaluation of Disability Inventory (PEDI-UG) scores at baseline were 33 (9, 54) and 36 (8,70) on the caregiver-led and therapist-led arms respectively. The scores improved to 54 (35, 90) and 67 (15, 116) on the caregiver-led and therapist-led arms respectively. The mean difference between the two groups was 3 (95% CI: -13.4, 19.4), indicating comparable gains across the two groups. The changes in the self-care, mobility and social functioning areas are illustrated in (Table 4).

**Table 4:** Pre- and post-implementation change in self-care, mobility and social functioning of the children (PEDI-UG)

Outcome	Before training		After training		Post-intervention Between-group differences 95%CI
	Caregiver-led arm Median[IQR] *n* = 39	Therapist-led arm training Median[IQR] *n* = 37	Caregiver-led arm Median[IQR] *n* = 37	Therapist-led arm Median[IQR] *n* = 37	
PEDI-total score	33[9,54]	36[8,70]	54[35,90]	67[15,116]	3(-13.4, 19.4)
PEDI-self-care	9[4,18]	7[4,18]	15[10,26]	15[6,30]	-1(-5, 3)
PEDI-mobility	4[1,14]	5[2,19]	6[4,18]	10[3,31]	1(-2.5, 4.5)
PEDI-Social	8[2,13]	5[2,15]	12[6,24]	14[3,27]	-1(-4.8, 2.8)
Caregiver assistance Self-care	2[1,8]	2[0,8]	8[6,9]	8[1,16]	-2(− 5.3, 1.3)
Caregiver assistance mobility	0[0,4]	0[0,7]	5[0,6]	5[0,11]	2(-1, 5)
Caregiver assistance social	0[0,5]	0[0,7]	5[3,7]	6[4,9]	1(-1.7, 3.7)

This table presents changes in the Paediatric Evaluation of Disability of Evaluation Inventory-Ugandan version (PEDI-UG) across the functioning domains of self-care, mobility, and social functioning before and after the training programme, comparing the caregiver-led and therapist-led arms. Data are shown as medians with interquartile ranges (IQR). Between-group median differences are reported alongside their 95% confidence intervals

Although the CASP allows for a total score, it was not calculated in this study as some domains were not applicable to all children, making a summary score potentially misleading. Instead, subscale-level changes were examined. Median scores across domains were modest, with wide interquartile ranges and confidence intervals that crossed zero. The wide interquartile ranges indicate considerable variability in scores within groups, while the 95% confidence interval crossing zero suggests no statistically significant difference between the groups. Table 5 presents score distributions and change estimates by domain.

**Table 5:** Pre- and post-implementation change in participation of the children (CASP)

Outcome	Before training		After training			Post-training Between-group differences 95%CI
	Caregiver-led arm Median[IQR]	Therapist-led arm Median[IQR]	Caregiver-led arm Median[IQR]	Therapist-led arm Median[IQR		
Home Participation	33.3[25,45.8] *n* = 24	37.5[25,54.2] *n* = 29	45.8[33.3,54.2] *n* = 24		45.8[37.5,62.5] *n* = 29	4.2(-5.1, 13.4)
Community participation	34.4[25, 40.6] *n* = 24	37.5[31.3, 50] *n* = 29	37.5[25,50] *n* = 24		43.8[31.3,50] *n* = 29	6.25(-5.2, 17.7)
School Participation	45[40,50] *n* = 5	40[25,55] *n* = 11	45[40,55] *n* = 7		50[50,60] *n* = 11	-7.5(-9.9, 24.9)
Home & Community living	20[15,20] *n* = 24	20[15,20] *n* = 29	20[15,20] *n* = 24		20[15,20] *n* = 29	0(-4.7, 4.7))

This table presents changes in the Child and Adolescent Scale of Participation (CASP) across the participation domains before and after the training programme, comparing the caregiver-led and therapist-led arms. Data are shown as medians with interquartile ranges (IQR). Between-group median differences are reported alongside their 95% confidence intervals

## Discussion

This feasibility randomised controlled trial demonstrated that a caregiver-led training programme for caregivers of children with cerebral palsy (CP) can be successfully delivered in a rural, resource-limited setting. The trial met key feasibility indicators, including high participant retention, full programme completion, and strong fidelity to the intervention protocol. These findings suggest that caregiver-led delivery is both practical and acceptable in this context, supporting the case for further investigation in a powered trial.

The high participant retention and programme completion rates may be attributed to the programme’s timing and format, which resembled other community-based interventions conducted by the CBO. Designing the programme to align with participants’ needs and the local context may have helped to sustain engagement. Additionally, transport support facilitated continued attendance despite geographic barriers. The future powered trial of this programme in this setting should involve offering it in the caregivers’ immediate communities (sub-centres of Tiyende Pamodzi), eliminating the need for transport costs and likely maintaining high retention rates. The provision of logistical support such as transport and refreshments is important for maintaining retention [12, 50].

Fidelity to programme delivery was maintained across both arms. All facilitators whether expert caregivers or physiotherapists delivered the seven modules as per protocol. This consistency in content delivery supports the validity of outcome patterns observed and reinforces that differences in facilitator background did not compromise intervention delivery. Implementation monitors played a crucial role in maintaining fidelity. Notably, expert caregivers delivered modules more efficiently due to their fluency in both local languages, illustrating a key contextual strength of the caregiver-led approach in rural, multilingual communities.

Exploratory outcome data showed similar patterns of change across both the caregiver-led and therapist-led groups. The study was not designed or powered to assess effectiveness and should not be interpreted as demonstrating equivalence or non-inferiority [51, 52]. Nevertheless, observed between-group differences in the main programme outcome (PedsQL-FIM) were small, suggesting that large differences between delivery approaches are unlikely, and these estimates can inform non-inferiority margin selection and sample size calculations for a future trial [53]. Improvements in other caregiver-level outcomes were encouraging, with self-efficacy gains notably higher in the caregiver-led group. This provides an important signal for outcome prioritisation and sample size planning in future trials, and may reflect the influence of peer modelling and experiential knowledge transfer unique to expert caregiver facilitation. These findings are consistent with peer-led programmes in LMICs, including Baby Ubuntu and Juntos, which highlight the role of shared lived experience in strengthening caregiver confidence and coping capacity [10, 14].

Child-level outcomes showed modest but positive improvements, similar to those reported in other caregiver skills training interventions, including the WHO CST and Baby Ubuntu trials [23, 44, 54]. The improvements in PEDI-UG scores support the potential developmental benefits of caregiver-delivered programmes. However, challenges were noted with the CASP tool, particularly in domains assessing school and community participation environments to which many children in this sample had limited access. This limitation echos findings from related LMIC trials and underscores the need for culturally and contextually appropriate outcome measures capable of capturing functional change in constrained settings [23].

In summary, this feasibility randomised controlled trial confirmed the practicality of implementing a caregiver-led training programme for caregivers of children with cerebral palsy in a rural Malawian context. The findings demonstrate strong programme fidelity, high retention, and comparable patterns of benefit to therapist-led delivery. These results justify progression to a fully powered trial to formally evaluate effectiveness and assess non-inferiority. Future trials should consider including alternative or adapted participation measures to better reflect the lived experiences of children with limited access to school and community activities.

### Strengths and limitations

This trial is among the first to evaluate the feasibility of a caregiver-led training programme specifically tailored for families of children with cerebral palsy in a low-resource setting. Including a therapist-led arm provided a valuable comparison to guide implementation refinement, while ensuring that all participants received a potentially beneficial intervention. Although between-group programme outcome comparisons were conducted, the study was not powered to determine non-inferiority. Rather, the findings offer preliminary estimates of variability and outcome patterns to inform the design of a future powered trial.

Several practical constraints informed the trial’s design and delivery. Changes in caregiving behaviours were not verified through video analysis due to logistical challenges; future studies may incorporate home-based video assessments to objectively capture behavioural changes. Although sessions were held on separate days, a small risk of contamination cannot be excluded due to individual-level randomisation within shared community settings. Although stratified randomisation was used based on GMFCS level, other participant characteristics (e.g., age, CP subtype, caregiver relationship) were not balanced between arms; these differences could have influenced engagement and responsiveness. Future trials could consider stratifying on multiple participant characteristics to improve balance or using cluster randomisation at the subcentre level to reduce contamination.

Future larger studies may also consider a waitlist-controlled crossover design to enable a non-intervention comparison period and strengthen attribution of intervention effects. Additionally, the self-efficacy and knowledge tools used in this trial were programme-specific and not psychometrically validated. While these tools were contextually relevant, their use limits generalisability. Future research should prioritise validated or standardised instruments where feasible.

Finally, as participants were affiliated with a community-based organisation, there is potential for social desirability bias. Broader inclusion of caregivers not linked to structured programmes may enhance the generalisability of future findings.

## Conclusion

This study demonstrated that the Malamulele Onward Carer-to-Carer-Training Programmme is feasible to implement in a low-resource setting, with high fidelity of delivery, strong participant retention, and promising patterns in caregiver and child outcomes. While the observed changes were encouraging, they should be interpreted as exploratory rather than conclusive. These findings support the potential of caregiver-led training to address critical service gaps in contexts with limited access to rehabilitation professionals.

The trial contributes to the growing global evidence base on task-sharing in rehabilitation and responds to calls for more inclusive, scalable, and sustainable models of care. The results offer practical insights for global health stakeholders seeking to strengthen family-centred services in resource-constrained settings and lay a valuable foundation for designing a fully powered trial to formally assess effectiveness and refine task-sharing approaches.

## Supplementary Information

Supplementary Material 1.

Supplementary Material 2.

Supplementary Material 3.

## Data Availability

The de-identified dataset supporting the conclusions of this article is available in the Zenodo repository at [https://doi.org/10.5281/zenodo.14013425](https:/doi.org/10.5281/zenodo.14013425) ,
