# Supplementary material for: Caregiver-led versus therapist-led training programme for caregivers of children with cerebral palsy in rural Malawi: a feasibility randomised controlled trial
Source: BMC Pediatr. 2026 Jul 7;26:1118. doi: 10.1186/s12887-026-07286-4 (PMC13733084; doi:10.1186/s12887-026-07286-4)
Supplement: Supplementary file 2 [file 12887_2026_7286_MOESM1_ESM.docx]

**SESSION MONITORING TOOL**

**Date:**

**Name of workshop:**

**Name of facilitator (s):**

**Length of session:**

**Number of participants:**

|  | **Session section name** | **Time allotted to the session** | **Remark** |
| --- | --- | --- | --- |
| **1** |  |  |  |
| **2** |  |  |  |
| **3** |  |  |  |
| **4** |  |  |  |
| **5** |  |  |  |
| **6** |  |  |  |

**How was today’s lesson? (Phunziro la lero linali bwanji?)**

**………………………………………………………………………………………………………………………………………………………………………………………………………………………………………………………………………………………………………………………………………………………………………………………………………………………………………………………………………………………………………………………………………………………………………………………………………………………………………………………………………………………………………………………………………………………………………………………………………………………………………………………………………………………………………………………………………………………………………………………………………………………….**

**What went well and why? (Ndi mbali ziti zaphunziro zayenda bwino? Chifukwa chiyani?)**

……………………………………………………………………………………………………………………………………………………………………………………………………………………………………………………………………………………………………………………………………………………………………………………………………………………………………………………………………………………………………………………………………………………………………………………………………………………………………………………………………………………………………………………………………………………………………………………………………………………

**What did not go well and why? (Ndi mbali ziti za phunziro sizinayende bwino? Chifukwa chani?)**

………………………………………………………………………………………………………………………………………………………………………………………………………………………………………………………………………………………………………………………………………………………………………………………………………………………………………………………………………………………………………………………………………………………………………………………………………………………………………………………………………………………………………………………………………………………………………………………………………………………………………………………………………………………………………………………………………………………………………………………………………………………….

**Other comments & observations / Ndemanga zina**

**(e.g., facilitator teaching style, learner participation etc /ndemanga pa kaphunzitsidwe komanso kutenga mbali kwa ophunzira):**

**…………………………………………………………………………………………………………………………………………………………………………………………………………………………………………………………………………………………………………………………………………………………………………………………………………………………………………………………………………………………………………………………………………………………………………………………………………………………………………………………………………………………………………………………………………………………………………………………………………………………………………………………………………………………………………………………………………………………………………………………………………………………**
