# Supplementary material for: Caregiver-led versus therapist-led training programme for caregivers of children with cerebral palsy in rural Malawi: a feasibility randomised controlled trial
Source: BMC Pediatr. 2026 Jul 7;26:1118. doi: 10.1186/s12887-026-07286-4 (PMC13733084; doi:10.1186/s12887-026-07286-4)
Supplement: Supplementary file 3 [file 12887_2026_7286_MOESM2_ESM.docx]

FIDELITY CHECKLIST

| MODULE | PART | Group 1 | Group 2 | Facilitator Signature | Monitor Sign |
| --- | --- | --- | --- | --- | --- |
|  |  |  |  |  |  |
| Module one | Introduction |  |  |  |  |
|  | Outcome description |  |  |  |  |
|  | What we know about CP |  |  |  |  |
|  | Sharing our stories |  |  |  |  |
|  | What is CP |  |  |  |  |
|  | How CP affects the body |  |  |  |  |
|  | How do we know that a child has CP |  |  |  |  |
|  | The different subtypes of CP |  |  |  |  |
|  | The different levels of CP |  |  |  |  |
|  | The different patterns of CP |  |  |  |  |
|  | Cause of CP |  |  |  |  |
|  | The outlook of CP -Lifelong condition |  |  |  |  |
|  | Schooling for children with CP |  |  |  |  |
|  | Take home message |  |  |  |  |
|  | Total session length:………….. |  |  |  |  |
| Module two | Introduction (Icebreaker) |  |  |  |  |
|  | Outcome description |  |  |  |  |
|  | Looking at CP as a way of life |  |  |  |  |
|  | How a child with CP may change over time |  |  |  |  |
|  | How change may be negative over time |  |  |  |  |
|  | How change may be positive over time |  |  |  |  |
|  | The importance of good positioning |  |  |  |  |
|  | Movement of the hip joint |  |  |  |  |
|  | Positioning the child in helpful ways |  |  |  |  |
|  | Importance of standing and standing position |  |  |  |  |
|  | Caring for a child with CP in helpful ways |  |  |  |  |
|  | Caring for a child who experiences stiff limbs |  |  |  |  |
|  | Caring for a child who experiences abnormal movements (dyskinesia) |  |  |  |  |
|  | How to use everyday activities to help our child |  |  |  |  |
|  | Dressing and undressing |  |  |  |  |
|  | Take home message |  |  |  |  |
|  | Total session length:…………… |  |  |  |  |
| Module three | Welcome remarks/Introduction and icebreaker |  |  |  |  |
|  | Description of learning outcomes |  |  |  |  |
|  | Understanding our child’s body |  |  |  |  |
|  | Preparing the child’s body for movement |  |  |  |  |
|  | Children who experience stiff limbs (spastic) |  |  |  |  |
|  | Moving, twisting and stretching |  |  |  |  |
|  | Good positioning for children with stiff limbs |  |  |  |  |
|  | Children who experience fluctuating tone (dystonia) |  |  |  |  |
|  | Good positioning for children with dytonia |  |  |  |  |
|  | Children who experience too much movement (choreoathetiod) |  |  |  |  |
|  | Good positioning for children with choreoathetosis |  |  |  |  |
|  | Ideas for children on levels 1, 2 and 3 |  |  |  |  |
|  | Take home message |  |  |  |  |
|  | Total session length:………………….. |  |  |  |  |
| Module four | Introduction/Icebreaker/ |  |  |  |  |
|  | Description of outcomes |  |  |  |  |
|  | Good positioning for feeding |  |  |  |  |
|  | Spoon feeding |  |  |  |  |
|  | Preparing the mouth/lips |  |  |  |  |
|  | Helping the child learn to chew |  |  |  |  |
|  | Helping the child feed self |  |  |  |  |
|  | Feeding using a cup |  |  |  |  |
|  | Helping a child who drools |  |  |  |  |
|  | Eating well (the right food) |  |  |  |  |
|  | Cleaning the child’s feed |  |  |  |  |
|  | Take home message |  |  |  |  |
|  | Total session length:……………… |  |  |  |  |
| Module five | Introduction/welcome remarks /Icebreaker |  |  |  |  |
|  | Description of outcomes |  |  |  |  |
|  | What is communication |  |  |  |  |
|  | Different ways of communication |  |  |  |  |
|  | Why it is difficult for a child with CP to communicate |  |  |  |  |
|  | How to help a child with CP to communicate |  |  |  |  |
|  | How to help a child with CP understand well |  |  |  |  |
|  | What can we communicate about ? |  |  |  |  |
|  | Communicating /taking about every day things |  |  |  |  |
|  | How we can improve the child’s communication skills |  |  |  |  |
|  | Teaching a child to use signs |  |  |  |  |
|  | Practicing communication skills |  |  |  |  |
|  | Take home message |  |  |  |  |
|  | Total session length:………….. |  |  |  |  |
| Module six | Introduction/icebreaker/ |  |  |  |  |
|  | Description of outcomes |  |  |  |  |
|  | What is play and how important is play? |  |  |  |  |
|  | Learning through play |  |  |  |  |
|  | How children play |  |  |  |  |
|  | Why it can be difficult for children with CP to play |  |  |  |  |
|  | How can we help children with CP to play? |  |  |  |  |
|  | Making play easy for children with CP |  |  |  |  |
|  | Making useful play things/toys for children with CP |  |  |  |  |
|  | Helping other children play with your child |  |  |  |  |
|  | What can we play with |  |  |  |  |
|  | Helping a child with CP play using both hands |  |  |  |  |
|  | Take home message |  |  |  |  |
|  | Total session length:……………. |  |  |  |  |
| Module seven | Introduction /Icebreaker |  |  |  |  |
|  | Description of learning outcomes |  |  |  |  |
|  | Visual problems |  |  |  |  |
|  | How can we know that the child cannot see |  |  |  |  |
|  | Helping a child with visual problems |  |  |  |  |
|  | How should we hold things |  |  |  |  |
|  | How to use colour differences |  |  |  |  |
|  | Using clear backgrounds to assist sight |  |  |  |  |
|  | Using brightness and contrast to assist vision |  |  |  |  |

KEY

| √ | Delivered |
| --- | --- |
| × | Not delivered |
